# Supplementary material for: T Regulatory Cell-Associated Tolerance Induction by High-Dose Immunoglobulins in an HLA-Transgenic Mouse Model of Pemphigus
Source: Cells. 2023 May 8;12(9):1340. doi: 10.3390/cells12091340 (PMC10177252; doi:10.3390/cells12091340)
Supplement: Supplementary file 1 [file cells-12-01340-s001.zip › cells-2359282-supplementary.pdf]

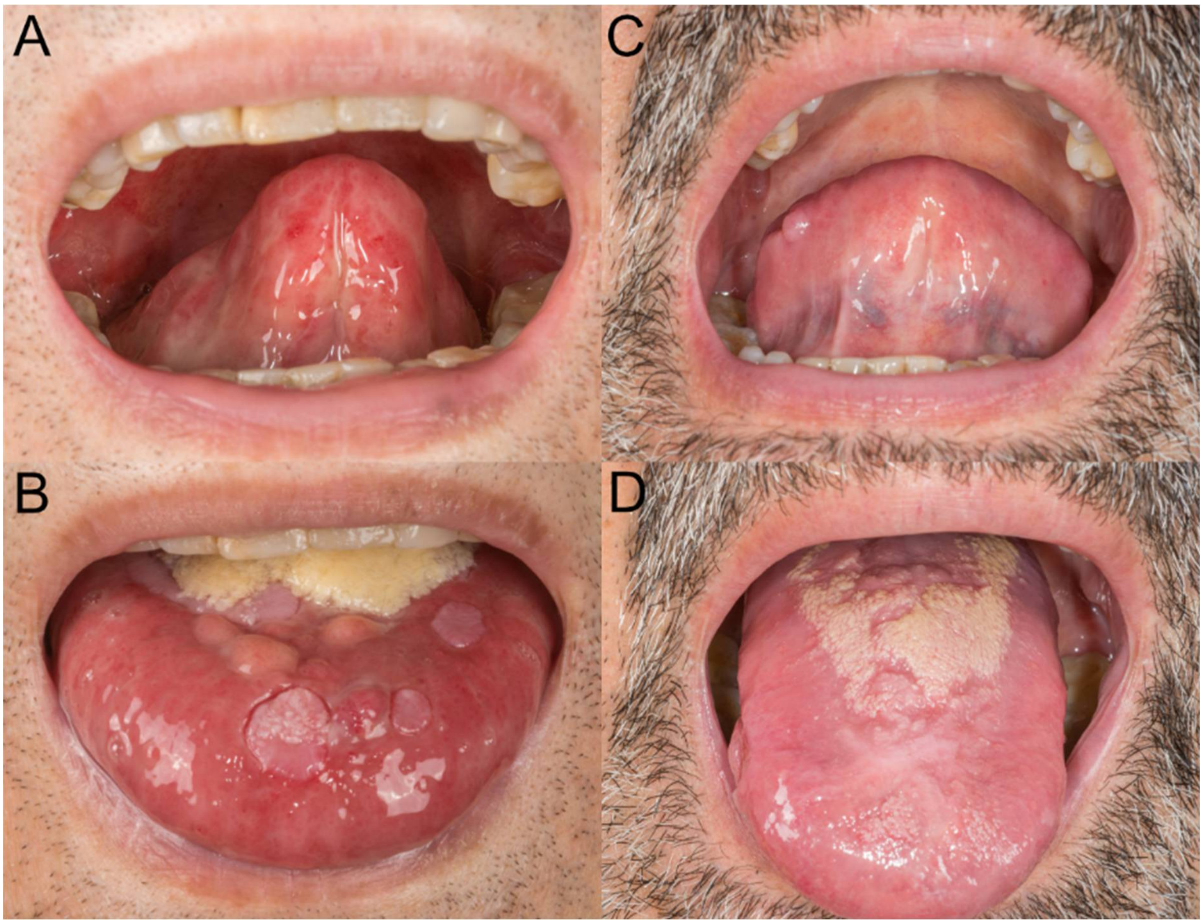

Figure S1. Treatment efficiency of IVIg in a patient with pemphigus vulgaris. Flaccid blisters formation and erosions of the tongue prior (A, B) and 7 month after IVIg treatment (C, D).

A

Group	number of IVIg applications	total amout of IVIg applied	length of experiment (days)	days between last treatment and analysis
A1	3	6 mg	21	5
A2	4	8 mg	35	12
A3	4	8 mg	70	47
B1	1	2 mg	35	5
B2	5	10 mg	63	5
B3	5	10 mg	77	18

B

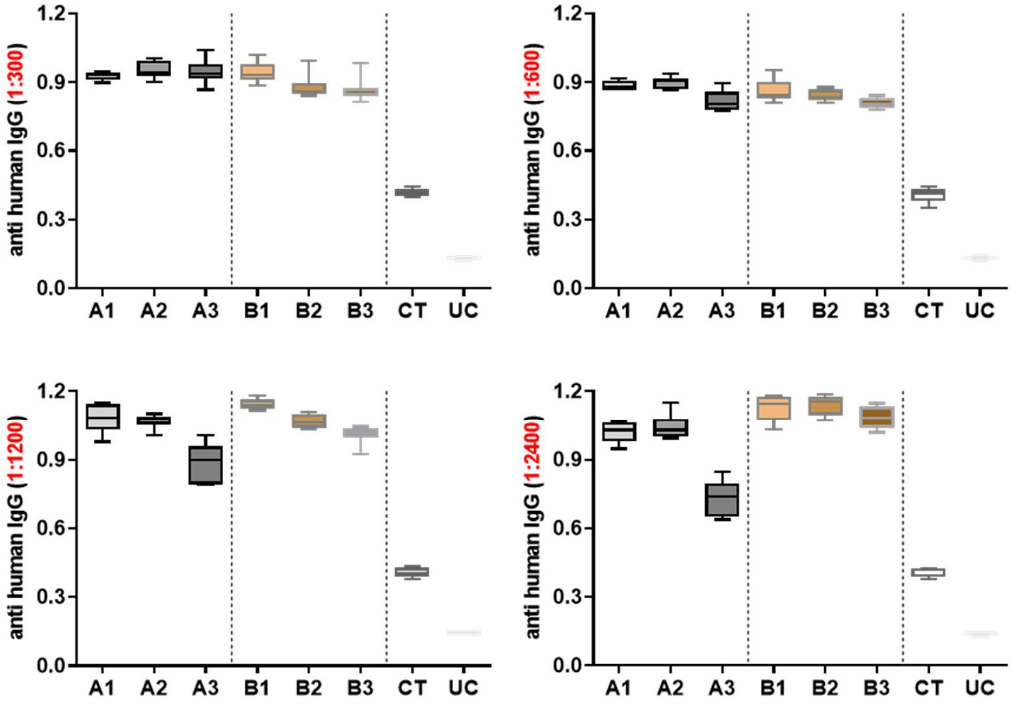

Figure S2. Summary of accumulative IVIg load in respective treatment groups and human IgG quantification by ELISA. (A) Group comparison regarding IVIg treatment. **(B)** Quantification of human IgG by ELISA in sera of treated mice at indicated dilutions displayed at OD_{405}. Error bars in graphs represent standard deviation, mean ±SD. n=6-8 mice per group. PBS immunized samples, control (CT). uncoated wells (UC).

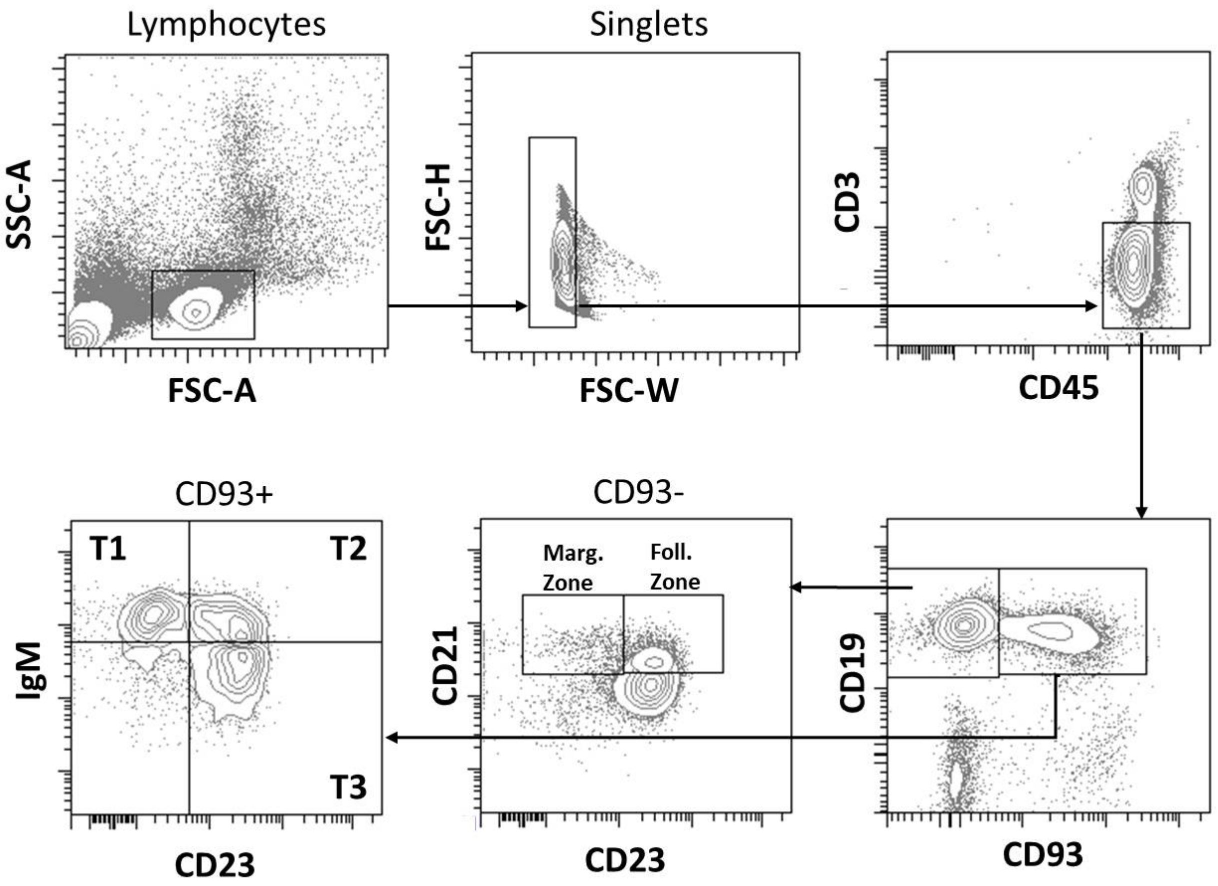

Figure S3. Representative display of B cell gating strategy for the detection of murine splenic B cell subsets. Marg. Zone, marginal zone B cells; Foll. Zone, follicular zone B cells.

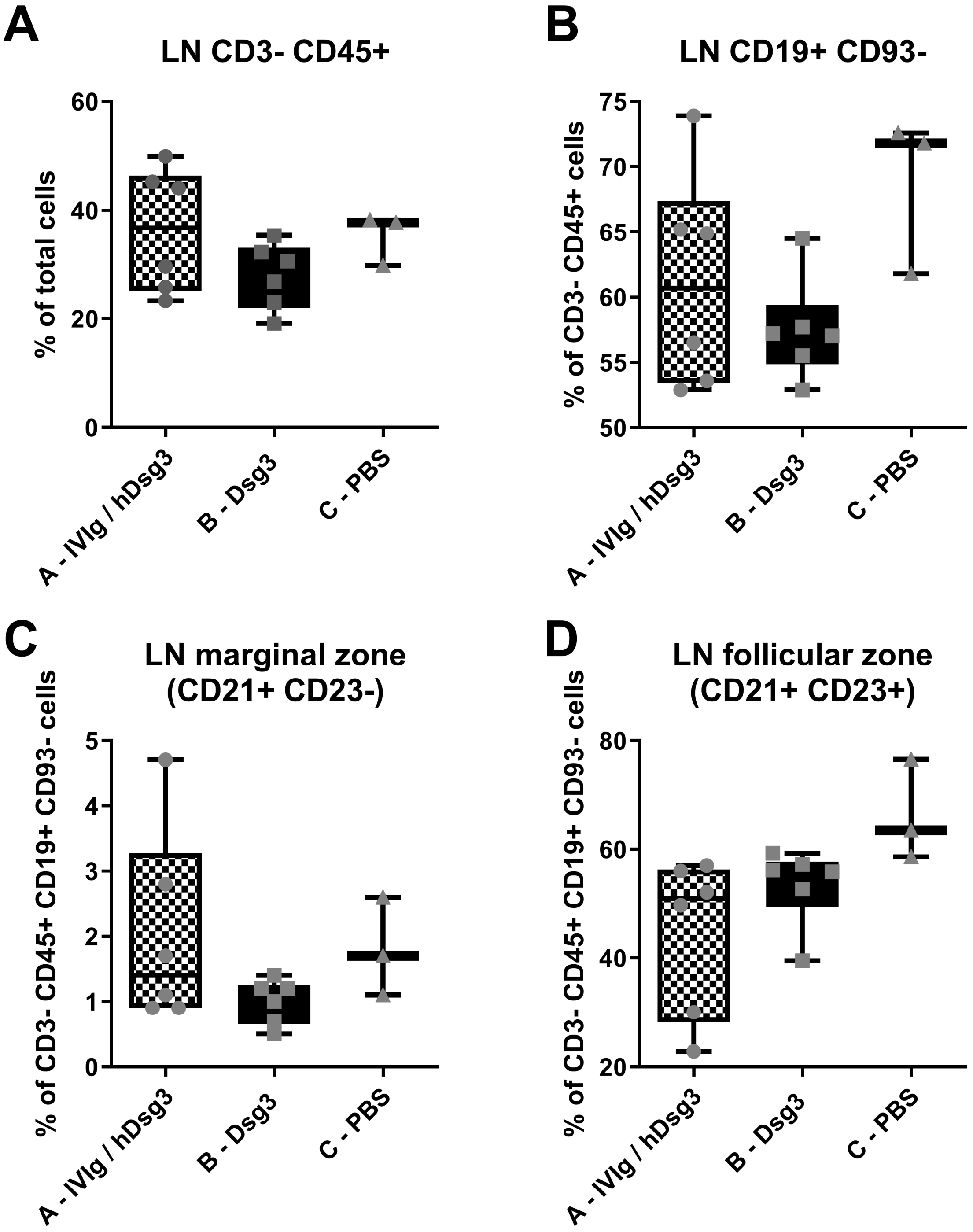

Figure S4. Representative display of experimental B cell subset analysis. Parent B cell population (CD3- CD45+ CD19+ CD93-) **(A, B)** was further stratified into marginal zone B cells **(C)** and follicular zone B cells **(D)**. LN, lymph nodes. n=3 (PBS) - 6 mice per group.
